# Drought prediction using artificial intelligence models based on climate data and soil moisture

**DOI:** 10.1038/s41598-024-70406-6

**Published:** 2024-08-24

**Authors:** Mhamd Saifaldeen Oyounalsoud, Abdullah Gokhan Yilmaz, Mohamed Abdallah, Abdulrahman Abdeljaber

**Affiliations:** 1 Department of Civil and Environmental Engineering, University of Sharjah, Sharjah, United Arab Emirates; 2 Department of Engineering, School of Computing, Engineering and Mathematical Science, La Trobe University, Melbourne, Australia; 3 Department of Civil Engineering, University of Ottawa, Ottawa, ON Canada

**Keywords:** Meteorological drought, Drought indices, Forecasting, Soft computing, Drought indicators, Environmental sciences, Hydrology, Engineering, Mathematics and computing

## Abstract

Drought is deemed a major natural disaster that can lead to severe economic and social implications. Drought indices are utilized worldwide for drought management and monitoring. However, as a result of the inherent complexity of drought phenomena and hydroclimatic condition differences, no universal drought index is available for effectively monitoring drought across the world. Therefore, this study aimed to develop a new meteorological drought index to describe and forecast drought based on various artificial intelligence (AI) models: decision tree (DT), generalized linear model (GLM), support vector machine, artificial neural network, deep learning, and random forest. A comparative assessment was conducted between the developed AI-based indices and nine conventional drought indices based on their correlations with multiple drought indicators. Historical records of five drought indicators, namely runoff, along with deep, lower, root, and upper soil moisture, were utilized to evaluate the models’ performance. Different combinations of climatic datasets from Alice Springs, Australia, were utilized to develop and train the AI models. The results demonstrated that the rainfall anomaly drought index was the best conventional drought index, scoring the highest correlation (0.718) with the upper soil moisture. The highest correlation between the new and conventional indices was found between the DT-based index and the rainfall anomaly index at a value of 0.97, whereas the lowest correlation was 0.57 between the GLM and the Palmer drought severity index. The GLM-based index achieved the best performance according to its high correlations with conventional drought indicators, e.g., a correlation coefficient of 0.78 with the upper soil moisture. Overall, the developed AI-based drought indices outperformed the conventional indices, hence contributing effectively to more accurate drought forecasting and monitoring. The findings emphasized that AI can be a promising and reliable prediction approach for achieving better drought assessment and mitigation.

## Introduction

Climate change triggers enduring shifts in climate patterns and is mainly triggered by greenhouse gas (GHG) emissions from anthropogenic activities. Such activities have resulted in approximately 1.0 °C of global warming above the preindustrial level, and this temperature change is projected to reach 1.5 °C by 2030^1^. This temperature rise has led to alterations in key climate parameters, such as humidity, precipitation, and wind speed. These alterations, particularly in precipitation, impact streamflow, resulting in extreme events involving droughts and floods^2^. Drought, defined as an extended precipitation-deficient period that causes inadequate surface water availability, is deemed one of the main consequences of climate change^3^. Research has indicated that water cycle intensification from GHG-induced global warming has elevated the likelihood and severity of surface aridity and droughts, with the frequency and duration of droughts expected to double in Africa and Asia^4–6^. Drought can lead to various devastating effects, e.g., soil erosion, water supply shortages, and crop destruction. Such impacts could potentially result in the displacement of around 700 million people by 2030^7^.

Urquijo and De Stefano^8^ addressed the definition of drought from a conceptual and operational perspective. Drought is a natural phenomenon typically categorized into four main classifications, namely, agricultural, hydrological, meteorological, and socioeconomic drought. The first three classifications address drought as a physical phenomenon, whereas the fourth classification tracks the effects of water insufficiency on human beings through socioeconomic systems. Agricultural drought is related to meteorological drought involving agrarian impacts due to precipitation scarcity and soil water deficits. Meteorological drought is determined according to the degree of aridity, while hydrologic drought occurs when the water supply becomes insufficient, especially in streams and groundwater^9^. On the other hand, socioeconomic drought relates the demand and supply of several commodities to drought. According to Danandeh Mehr et al.^10^, each classification entails identifying a set of parameters: (1) soil moisture and evaporation stress for agricultural drought, (2) streamflow shortages for hydrological drought, (3) evaporation and precipitation for meteorological drought, and (4) water demand and storage resilience for socioeconomic drought.

Drought can present various spatiotemporal characteristics, including severity, magnitude, intensity, duration, and geographic extent^11^. Several drought indices were proposed to quantify and analyze the various aspects of drought. Drought indices serve as essential instruments for the empirical assessment and analysis of drought characteristics^12^. They are employed to delineate and scrutinize the specifics of drought across diverse hydrological landscapes, providing a standardized measure to compare drought severity in different regions.^13^. They are based on individual and/or multiple hydrometeorological variables, such as streamflow and precipitation, which are utilized to analyze the impacts of drought^14^. Multiple conventional indices were established to manage drought impacts. However, there is no single index capable of capturing and conveying the severity and intensity of such an event^15^. Initially, negative precipitation anomalies were taken as an index to represent drought^11,16^, but they could not address the influence of drought on hydrology and agriculture. In this regard, alternative indices have been introduced, such as the standardized precipitation index (SPI), standardized precipitation evapotranspiration index (SPEI), and Palmer drought severity index (PDSI).

The PDSI was widely used in the late twentieth century^17,18^. It considers streamflow soil, moisture, potential evapotranspiration, and antecedent precipitation. Nevertheless, despite its utility, PDSI is limited by a built-in time scale that can delay its response to diminishing drought conditions, making it less sensitive to rapid changes in drought status^14^. On the other hand, the SPI has simple definitions and requires fewer variables. The SPI utilizes multiscale attributes to be employed for simultaneous monitoring of moisture settings in different regions^11^. However, it focuses exclusively on precipitation without considering the impact of temperature, thus restricting its application in the context of global warming^14^. To overcome this limitation, the SPEI was developed, which addresses drought severity by establishing a balance between water demand and supply according to temperature and the difference between evapotranspiration and precipitation^19^.

Several studies have assessed the efficiency of various drought indices over the past few decades. For instance, Yang et al.^11^ examined the regional suitability of different meteorological drought indices in China, including the PDSI, modified PDSI, self-calibrating PDSI (scPDSI), SPI, SPEI, and surface wetness index. The results concluded that the scPDSI was the most appropriate index for reflecting the specific Chinese climatic conditions. Nevertheless, the scPDSI slightly decreased the value range compared to that of the PDSI; hence, the process of classifying wet and dry conditions should be modified accordingly. Moreover, Wable et al.^20^ evaluated the performance of Departure from Normal, the effective drought index, the SPI, the SPEI, and the reconnaissance drought index (RDI) in western India. The indices were computed for different timescales (1, 3, 6, 9, and 12 months) by utilizing datasets acquired from four climate stations over 25 years. The analysis demonstrated that SPEI of 9 months duration was the most suitable index in semiarid conditions. Another research evaluated the SPI and SPEI in the Tigray Region of Northern Ethiopia^21^. The findings showed that those indices demonstrated a fair level of agreement. Albeit the extensive use of conventional drought indices, their performance is highly sensitive to climatic conditions and changes substantially based on their input parameters. Furthermore, these methods sometimes produce unreliable outcomes when applied to different regions^22^. Those drawbacks have induced a shift toward applying soft computing techniques due to their minimal inputs, ease of implementation, and relatively low computational costs.

Several recent studies were focused on adopting various soft computing tools for drought prediction and monitoring. Malik et al.^23^ predicted the SPI at various time scales in India by developing a fuzzy logic model: coactive neuro-fuzzy inference system (CANFIS). The findings demonstrated that CANFIS was more suitable for predicting the SPI than classic regression models and artificial intelligence (AI). Moreover, Khan et al. (2020) tested a hybrid model containing wavelet transformation, an autoregressive integrated moving average technique, and an artificial neural network (ANN) to predict future droughts in Malaysia^24^. Drought indices such as the standard index of annual precipitation and SPI were utilized to calculate the historical drought events. The results indicated that the hybrid model exceeded a standalone ANN with coefficients of determination (R^2^) of 0.914 and 0.423, respectively. Furthermore, Mehr et al.^10^ established a hybrid model consisting of a convolutional neural network and a long short-term memory network for short-term prediction in Turkey. This model predicted the SPEI with a root mean square error (RMSE) between 0.043 and 0.075.

Despite the proven feasibility of soft computing in drought assessment and monitoring, these models have drawn little attention in drought evaluation research. Moreover, only a few studies compared the performance of different machine learning algorithms. The present study addresses those research gaps by applying various AI models to establish a new meteorological drought index. Moreover, this study employs drought indicators (e.g., root, deep, upper, and lower soil moisture and runoff) to assess the performance of the soft computing-based drought indices. The specific objectives of this study were to (1) evaluate the efficacy of the conventional indices through different drought indicators, (2) establish a new AI-based drought index and assess its ability to forecast drought, and (3) conduct a comparative assessment among several AI-based drought indices. This research introduces a novel contribution to the literature by integrating the best-performing conventional drought indices with the soil moisture measurements to develop an enhanced training dataset for the development of the AI-based drought indices. The proposed modeling framework was applied to predict drought indices in Australia, which is considered the driest inhabited continent worldwide. This study shall provide decision-makers with a robust approach to enhancing the accuracy of drought assessments toward better drought management and monitoring.

## Study area

**Figure 1:**
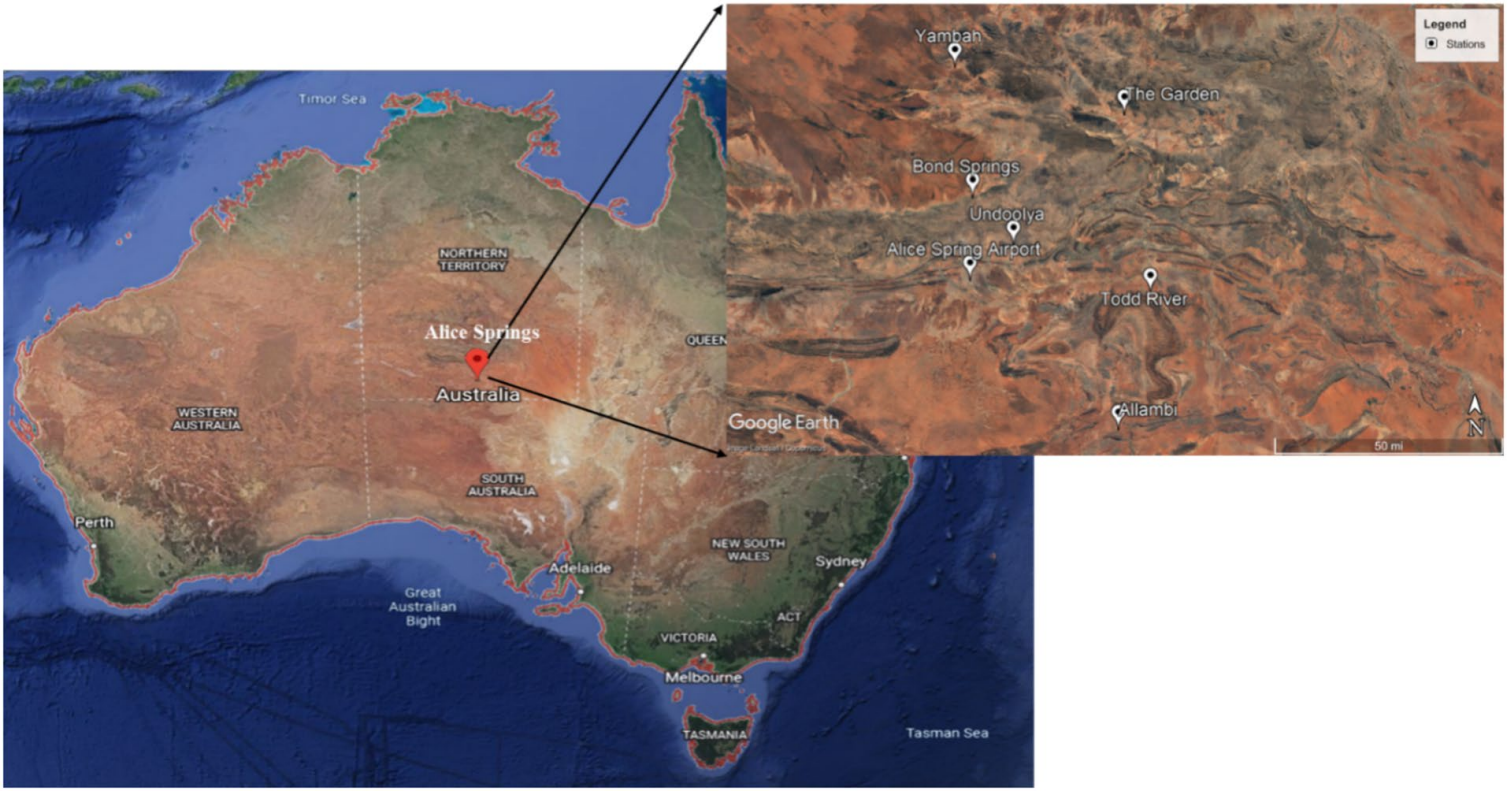
Satellite images for the locations of Alice Springs and the chosen stations in Australia (by Google Earth).

Alice Springs, located in the center of the Australian desert (23.70 °S latitude and 133.88 °E longitude), was selected as the study area. Figure 1 depicts the study area used in this research, obtained from Google Earth (version 6.0.2.2074). Data were obtained from seven meteorological stations in Alice Springs and utilized in the development and assessment of the drought index. Table 1 lists the seven meteorological stations coordinates. The monthly precipitation rate, mean, and maximum temperature data between 1985 and 2020 were acquired from the Australian Bureau of Meteorology (BoM). In addition to the aforementioned parameters, root, deep, upper, and lower soil moisture and runoff were utilized as drought indicators. These indicators were obtained monthly for all examined stations for the period of 2005–2020.

**Table 1:** Elevation and location of the studied meteorological stations in Alice Springs.

Meteorological stations	Elevation (m)	Latitude (S)	Longitude (E)
Alice Springs Airport	546	23° 48′ 00″	133° 53′ 24″
Yambah	700	23° 07′ 48″	133° 50′ 24″
Undoolya	558	23° 41′ 24″	134° 02′ 24″
Todd River	427	23° 50′ 24″	134° 30′ 36″
The Garden	678	23° 16′ 48″	134° 25′ 12″
Bond Springs Homestead	764	23° 32′ 24″	133° 55′ 12″
Allambi	366	24° 16′ 12″	134° 24′ 00″

## Methodology

**Figure 2:**
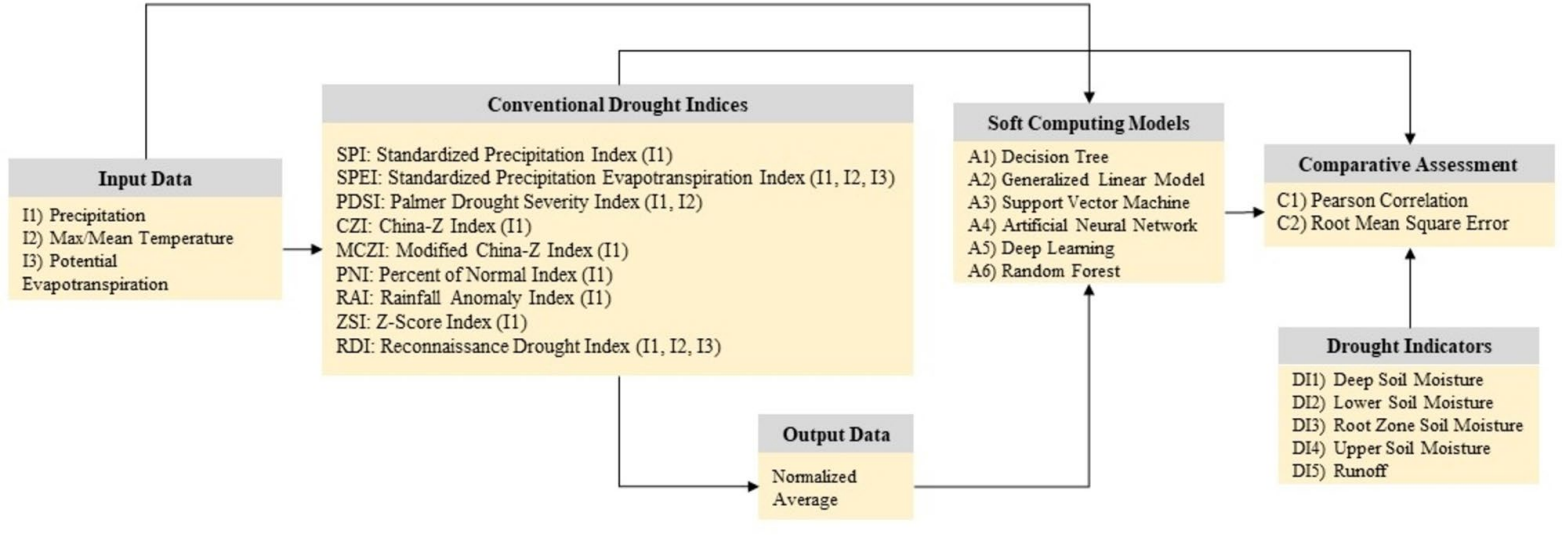
Schematic diagram of the methodological framework of this study.

This research originated to develop a novel AI-based drought index for drought assessment and monitoring based on various machine learning algorithms. The methodological framework for developing the drought index is illustrated in Fig. 2.

A set of climatic data (maximum temperature, precipitation, and potential evapotranspiration (PET) data), along with multiple drought indicators: (1) DI1: deep soil moisture, (2) DI2: lower soil moisture, (3) DI3: root zone soil moisture, (4) DI4: upper soil moisture, and (5) DI5: runoff, were collected from the seven meteorological stations as inputs for the AI models. The data quality check was conducted to remove errors, duplications, and/or inconsistencies. Moreover, missing data entries were imputed using linear interpolation. Notably, maximum temperature data were sourced from four stations situated around the study area (Alice Springs Airport, Curtin Springs, Kulgera, and Jervois station). In contrast, precipitation records were acquired from the seven mentioned stations. To reconcile this, temperature datasets from the four stations were transformed to match all seven stations using the inverse distance weighting (IDW) method. Additionally, I3 was computed using the Thornthwaite method due to its minimal data requirement, including average temperature and latitude^25^.

The following procedures were applied to calculate drought indices using conventional methods with the R, Dmap, and Drinc software. Following the conventional methods for estimating the indices, a Pearson correlation analysis was used to compare the results of the conventional models with the drought indicators. The correlation between the outputs from the drought indicators and conventional models was utilized to develop and validate AI-based drought assessment indices using different machine learning algorithms.

### Conventional drought indices

The present study aims to evaluate the drought severity and duration through several conventional drought indices, obtained from the Alice Springs meteorological stations. The conventional meteorological drought indices applied in this research included the SPI, SPEI, PDSI, percent of normal index (PNI), China-Z index (CZI), modified China-Z index (MCZI), rainfall anomaly index (RAI), Z score index (ZSI), and RDI. In general, the range of each index varies significantly, in which negative and positive values denote dry and wet conditions, respectively; except for PNI, which has a range from 40 to 120. Information about the theory and ranges of each drought index can be found in the supplementary files.

#### Standardized precipitation index

The SPI was first developed in 1992 based on drought frequency, duration, and timescale. In terms of computation, the model relies on a single parameter, i.e., precipitation. This index has several advantages, including its flexibility to be calculated over a range of timeframes and its spatial consistency. Such a function allows for comparisons among various locations under different climate conditions. The SPI was calculated using estimated precipitation probabilities for a given timescale and then converted into an index. For any selected location, the SPI was calculated using long-term precipitation records. The obtained records were transformed from a probability distribution and fitted to a normal distribution model. The SPI was calculated using the following probability density function based on precipitation data fitted by gamma distribution ($g\left({x}\right)$):

1 $$g\left({x}\right)= \frac{1}{{\beta }^{\alpha }\Gamma \left(\alpha \right)}{{x}}^{\alpha -1}{e}^{-{x}/\beta }: {x}>0; \beta >0$$

where *β* is the scale parameter,* α* is the shape parameter, *Γ(α)* is the gamma function, and *x* is the amount of precipitation (mm).

#### Standardized precipitation evapotranspiration index

The SPEI was calculated based on the PET data using the Thornthwaite method, as it requires minimal data (i.e., average temperature and latitude) compared to other techniques^25^. The computations for the PET and SPEI can be found in the supplementary files.

#### Palmer drought severity index

The PDSI is a method of comparing moisture conditions in different locations and between different months based on standardized measurements of moisture conditions. An analysis of the PDSI is based on the precipitation, temperature, and soil properties. Based on the inputs, such as evapotranspiration, soil recharge, runoff, and moisture loss from the surface layer, the water balance equation can be constructed. Moreover, the PDSI represents the drought history spatially and temporally. Although the PDSI is designed for agriculture applications, the model does not accurately reflect the effects of prolonged droughts on hydrology. The PDSI was computed based on the methodology developed by^26^.

#### Percent of normal index

The PNI is used to classify droughts and determine the severity of meteorological events. The PNI was found to be efficient in evaluating the drought conditions for individual seasons as well as a specific region. Additionally, this index can be calculated on different time scales, such as monthly, seasonal, and annual basis. The PNI was calculated as follows:

2 $$PNI= \frac{{P}_{i}}{P} x 100$$

where *P*_*i*_ is the precipitation (mm) with time increment *i* and *P* is the normal precipitation (mm) for the selected time frame.

#### China-Z index and modified China-Z index

As a replacement for SPI, the CZI was developed by the National Climate Center of China in 1995 ^27^. The CZI was calculated as follows:

3 $$\text{CZIij}= \frac{6}{Csi}\times {\left(\frac{\text{Csi}}{2}\times \varphi tj+1\right)}^{1/3}-\frac{6}{Csi}+\frac{\text{Csi}}{6}$$

where *CZI*_*ij*_ stands for the corresponding CZI of the present month (J) for time period *i, i* represents the time scale of interest, *j* is the present month, *C*_*si*_ is the skewness coefficient, and *φ*_*tj*_ is the standardized variation. Additionally, the MCZI was calculated by substituting the median precipitation for the mean precipitation in the same equation.

#### Rainfall anomaly index

The RAI is a drought index that measures deviations of monthly or seasonal rainfall from the long-term average. The methodology involves calculating the standardized anomalies by comparing observed precipitation data against historical mean values. Positive and negative anomalies are quantified, providing an indication of wet and dry periods, respectively. An average of the ten highest values creates a threshold for the positive anomaly, and an average of the ten lowest values creates a threshold for the negative anomaly. The thresholds were calculated as follows:

4 $$RAI= \pm 3\times \left[\frac{(p-\overline{p })}{\overline{m }-\overline{p} }\right]$$

where *p* is the annual precipitation (mm), *p* refers to the long-term average precipitation (mm), and *m* is the mean of the ten highest/ lowest values of *p* for the positive/negative anomalies.

#### Z score index

The ZSI estimates the monthly moisture anomaly by assessing the deviation from normal moist conditions in a given month. Despite the similarities between the ZSI and SPI, the ZSI does not require fitting the precipitation data to gamma and Pearson type III distributions. The following equation was used to calculate ZSI:

5 $$ZSI= \frac{Pi-\overline{P}}{SD }$$

where *P* represents the average monthly precipitation (mm), *P*_*i*_ is precipitation for a specified month (mm), and *SD* refers to the standard deviation of a time series (mm).

#### Reconnaissance drought index

The RDI is based on the measured precipitation and PET, where it is a correlation of RDI initial value (α_k_), standardized RDI (RDI_st_), and normalized RDI (RDI_n_). A composite cumulative distribution function was used to calculate the RDI, which incorporates the probability of zero precipitation and the probability of gamma cumulative precipitation. The α_k_ was presented as an aggregated value for each month, season, and year, which is typically calculated for the year (i) and time basis j ( in months) according to the below equation:

6 $${{\alpha }}_{k}^{(i)}=\frac{\sum_{j=1}^{k}{P}_{ij}}{\sum_{j=1}^{k}{PET}_{ij}}$$

where *P*_*ij*_ is the precipitation and *PET*_*ij*_ is the potential evapotranspiration of month j for the hydrological year i. The RDI_st_ was calculated by fitting the gamma probability density function and the frequency distribution of α_k_. Given the assumption that α_k_ follows a lognormal distribution, RDI_st_ was calculated as follows:

7 $${RDI}_{st}^{i}=\frac{{y}^{(i)}-\overline{y}}{{\widehat{\sigma } }_{y}}$$

where ${y}^{(i)}$ is the ln (${{\alpha }}_{k}^{(i)}$), $\overline{y }$ is the arithmetic mean, and ${\widehat{\sigma }}_{y}$ refers to the standard deviation. The RDI_n_ was calculated as follows:

8 $${RDI}_{n}=\frac{{{\alpha }}_{k}^{(i)}}{\overline{{{\alpha } }_{k}}}-1$$

where $\overline{{{\alpha } }_{k}}$ represents the average of α_k_ values for the *n* years.

### Soft computing models

Several AI models, such as decision tree (DT), generalized linear model (GLM), support vector machine (SVM), artificial neural network (ANN), deep learning (DL), and random forest (RF), were used in this study for drought assessment. Hyperparameter tuning was conducted in RapidMiner using grid search to find the optimal set of parameters for each model. The tuned parameters were ANN: alpha, hidden layer sizes, max iteration, number of iterations, 2) RF: number of estimators, maximum depth, maximum features, minimum samples leaf, and minimum samples split.

#### Decision trees

DTs are efficient classification and regression white-box models that divide datasets into multiple subgroups. These models perform prediction by learning decision rules from the input parameters. The trees are branched through a recursive binary operation to minimize the sum of the squared deviations from the mean of the split parts. They are highly competent in managing data with different scales and nonlinearity. Furthermore, this model is capable of processing both discrete and continuous data types, incorporating an imputation technique where missing values are substituted with their mean or median values. Additionally, the model is adept at identifying the most relevant factors for modeling without the necessity for extensive hyperparameter tuning. Consequently, a DT was applied in the current research. More information can be found in^28^.

#### Generalized linear model

A GLM is a supervised machine learning algorithm that incorporates linear and logistic regression, where the data are fitted using the maximum likelihood approach. GLM develops a linear relationship between the response and the predictors despite having a nonlinear primary interaction mechanism. This is achieved by applying a link function to correlate the response with a linear model. The error distribution of the response follows an exponential distribution, e.g., a Poisson, binomial, or gamma distribution, as opposed to linear regression models. Furthermore, GLMs are effective in dealing with models possessing a constrained number of nonzero parameter predictors. GLM can be represented mathematically through the following equation:

9 $${Y}_{i}={\gamma }_{0}+{\gamma }_{1}{x}_{i1}+{\gamma }_{2}{x}_{i2}+\dots +{\gamma }_{n}{x}_{in}$$

where ${Y}_{i}$ is the predicted dependable variable, i.e., water consumption, *i* is the number of months, ${\gamma }_{0}$ is a constant model parameter, ${\gamma }_{i1}, {\gamma }_{i2}\dots {\gamma }_{in}$ and ${x}_{i1}, {x}_{i2}\dots {x}_{in}$ are the regression coefficients and independent variables, respectively, for the *i*th observation having *n* features.

#### Support vector machine

An SVM is an AI-based model initiated by a statistical learning theory. This method provides adequate generalization on a constrained number of learning patterns by adopting the inductive structural risk minimization principle. SVM utilizes a kernel function to obtain expert knowledge regarding the examined phenomenon to reduce the model complexity, hence increasing its forecasting accuracy^29^. The kernel function is considered a weighting function employed for nonparametric predictions. It can be modeled in linear, quadratic, and cubic forms. The mathematical kernel function of an SVM can be demonstrated in the equation below:

10 $$k(x,y)={\left[1+(x,y)\right]}^{p}$$

where *k* is the kernel function and *p* is a parameter assigned by the user to determine the order of the function.

#### Artificial neural network

An ANN is a computational method that mimics the functional behavior of a biological nerve cell in terms of processing information by connecting the inputs and outputs in an arranged aspect^30^. The structure of a typical ANN consists of neurons (processing units), connection weights, biases, and multiple layers. Conventional ANNs include one or more hidden layers, with neurons in each layer fully connected to every neuron in the subsequent layer. This architecture allows for the assignment of distinct weights and biases that facilitate the inter-neuron connections. ANNs offer various advantages over other models due to their robustness in interpreting complex, nonlinear data with high degrees of fluctuation.

A multilayer perceptron (MLP) was utilized in the present study to predict drought. An MLP is a feed-forward ANN that maps a series of inputs onto a set of appropriate outputs. The input data is fed into the input layer and progresses through the network, moving to all connected neurons in subsequent layers. An MLP employs backpropagation alongside the stochastic gradient descent optimization algorithm to train the network. During this process, the weights are adjusted iteratively until the network predicted values closely align with the actual observed values. In this study, an MLP with 300, 150, and 150 neurons in the input, output, and middle layers, respectively, was implemented. The value of each neuron in the hidden layer is determined by the weighted sum of its inputs, which is articulated in the following equation.

11 $${z}_{j}= \sum_{i=0}^{n}{w}_{ij}{x}_{i}-{b}_{j}$$

where *z*_*j*_ is the weighted sum, *n* is the number of inputs, *w*_*ij*_ is the weight of the input *x*_*i*_, and *b*_*j*_ is the bias value for the *j*th neuron. The deployed model used a constant learning rate of 0.001 and a stochastic gradient-based optimizer in tuning the squared loss^31^.

#### Deep learning

A DL model is considered a generalized form of a large neural network. It applies backpropagation to train a multilayer feedforward ANN via a stochastic gradient descent optimization algorithm. Its network can encompass several hidden layers comprised of neurons with tanh, rectifier, and maxout activation functions. DL offers various advanced attributes, particularly momentum training, an adaptive learning rate, and multiple regularization methods, which increase the forecasting accuracy of this approach. Each computational node trains the global model parameters on its local data with several asynchronous threads. Moreover, they add to the global model on a regular basis by performing model averaging throughout the network. More information on the development and application of DL models can be found in^32^.

#### Random forest

An RF is composed of a multitude of decision trees, each constructed by fitting random subsets of the dataset, and the collective output of these trees determines the final result. This method's inherent randomness in feature selection aids in preventing the model from overfitting. RFs are often chosen for ensemble modeling due to their proficiency in managing nonlinear data. They are capable of tackling large datasets with thousands of input variables and are versatile enough to address both regression and classification challenges. However, RF models involve intricate computations and may not yield precise predictions when dealing with data containing extreme values. The construction of a tree within the forest involves the random selection of data samples and the assignment of a specific number of features and observations to train the forest, allowing the trees to develop to their maximum depth.

### Drought indicators

Drought indicators are pivotal for the global assessment and management of drought conditions. The Australian Water Resources Assessment model, a joint initiative by the BoM and the Commonwealth Scientific and Industrial Research Organization, has developed multiple drought indicators based on various climatic data^33^. Soil moisture data were utilized in this study to assess the effectiveness of drought indices, as they are used to deduce drought events as early as possible^34^. The drought indicators applied in this study: (1) DI1: deep soil moisture, (2) DI2: lower soil moisture, (3) DI3: root zone soil moisture, (4) DI4: upper soil moisture, and (5) DI5: runoff, which provides a modeled estimate from a small, undisturbed watershed.

### Evaluation criteria

The evaluation criteria followed in this study were based on the Pearson correlation, the RMSE, and R^2^. The Pearson correlation coefficient is considered a statistical tool that determines the linear relationship between two parameters^35^. It was utilized in multiple applications, e.g., data analysis, noise reduction, and time-delay estimation. Pearson correlation was used to compare the outcomes of the conventional models with the drought indicators and validate the outputs of the soft computing models.

12 $$PCC=\frac{n\sum {x}_{i}{y}_{i}-\sum {x}_{i}\sum {y}_{i}}{\sqrt{n\sum {x}_{i}^{2}-{(\sum {x}_{i})}^{2}}\sqrt{n\sum {y}_{i}^{2}-{(\sum {y}_{i})}^{2}}}$$

where *PCC* is the Pearson correlation coefficient, *n* is the number of data points, *i* is the data point ranges from 1 to *n*, ${x}_{i}$ is the *i*th value of variable *x*, and ${y}_{i}$ is the *i*th value of variable *y*. In addition, the RMSE and R^2^ were the criteria utilized to assess the performance of the models. The former is sensitive to prediction errors, particularly for peak data values, serving as a measure of the models' accuracy, whereas the latter is indicative of the degree of agreement between the predicted and observed values^36^.

## Results and discussion

The current study applied several drought indices and correlated their output with multiple indicators to evaluate drought. A novel meteorological drought index was developed in this study by implementing various AI-based models and validating their outcomes via their correlations with various drought indicators to be applied for future drought monitoring.

### Data processing and analysis

**Figure 3:**
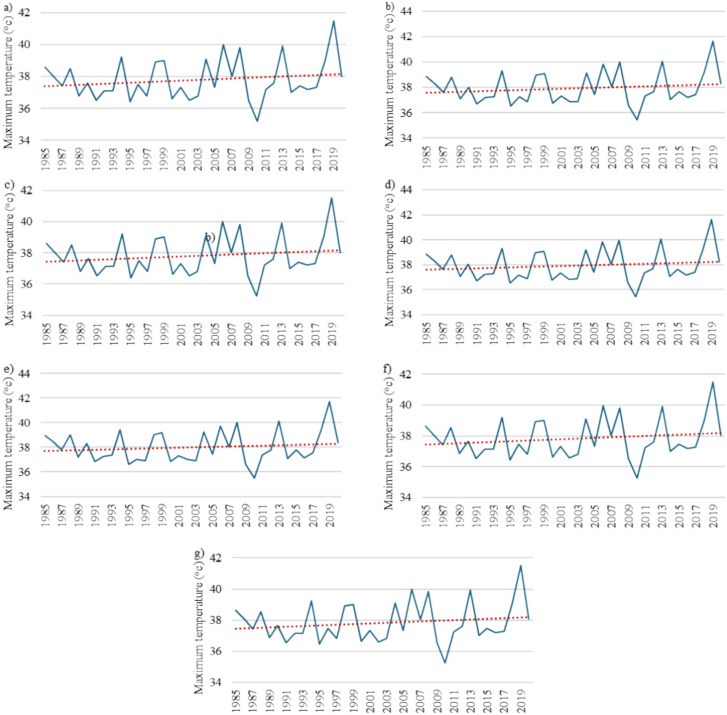
Temporal variations in the maximum temperature between 1985 and 2020 in the study area: (**a**) Alice Springs Airport, (**b**) Yambah, (**c**) Undoolya, (**d**) Todd River, (**e**) Garden, (**f**) Bond Springs Homestead, and (**g**) Allambi.

**Figure 4:**
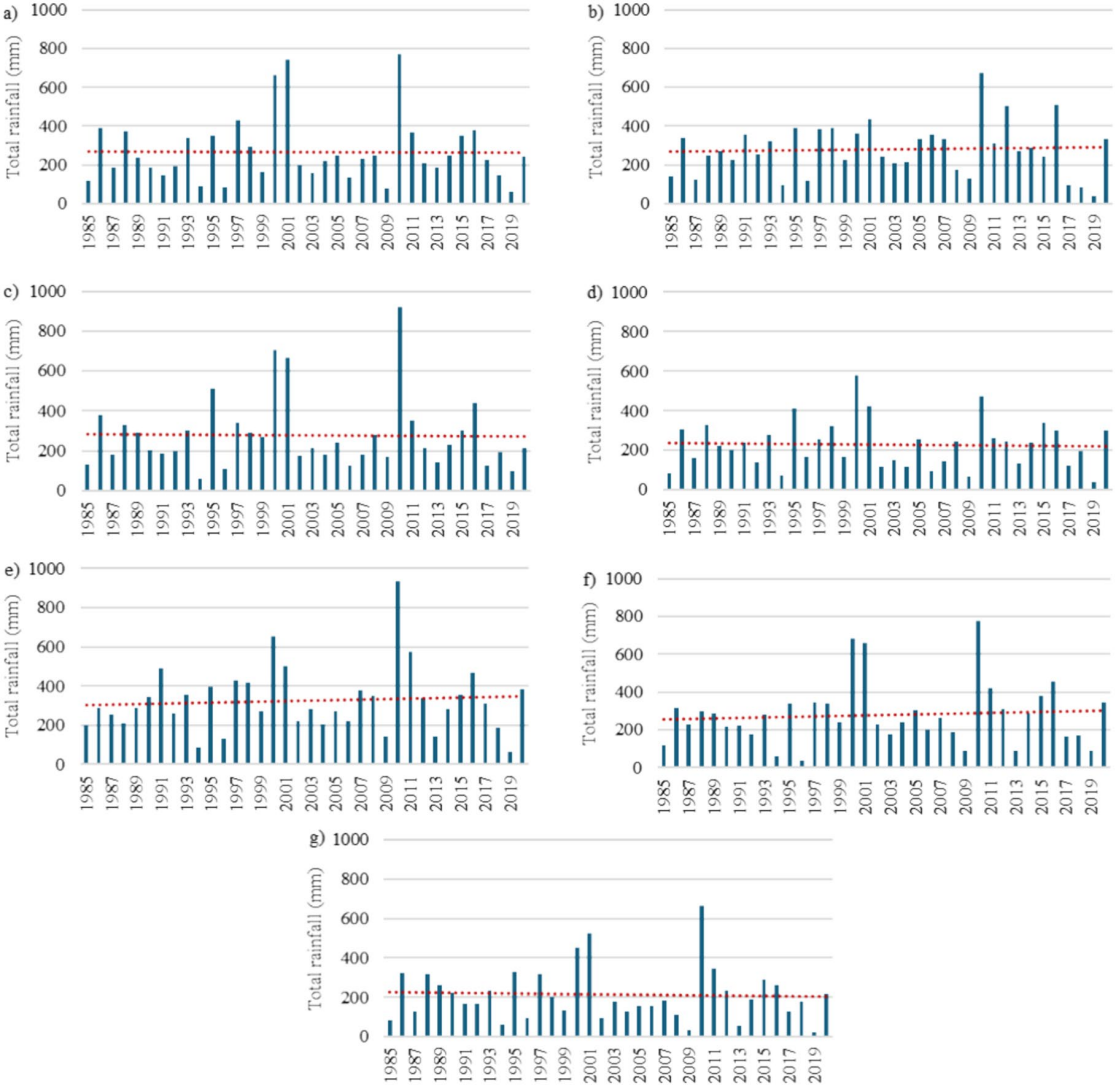
Temporal variations in rainfall between 1985 and 2020 in the study area: (**a**) Alice Springs Airport, (**b**) Yambah, (**c**) Undoolya, (**d**) Todd River, (**e**) Garden, (**f**) Bond Springs Homestead, and g) Allambi.

This study focused on 36 years of monthly data (1985–2020) compiled from BoM, Australia. Figures 3 and 4 show the temporal variations in the maximum temperature and rainfall at the selected stations in the study area, respectively. The collected data had a small number of gaps that needed to be properly filled prior to conducting the analysis; this was carried out through linear interpolation, one of several methods that were tested using the SPSS software. The rainfall data was acquired from seven stations, whereas the maximum and minimum temperature data was compiled from four stations. The only common station between the two sets of historical records was Alice Springs Airport. The IDW interpolation approach was utilized to collect the temperature data at the same seven stations as those that provided the rainfall data. As shown in Fig. 3, the maximum temperature ranged between 15 and 45 °C. The overall trend, represented by the red line, expresses the gradual increase in the recorded maximum temperatures. On the other hand, Fig. 4 displays the sharp fluctuations in the measured quantities of rain at the seven stations. In general, the wettest years were 1999 and 2000. However, there was no clear increasing or decreasing trend in the compiled rainfall data.

The adopted methodology in this study carried out drought evaluation through several drought indices, and then their outcome was correlated with selected indicators. The historical records of five drought indicators, DI1, DI2, DI3, DI4, and DI5, were collected for the same rainfall stations only from 2005 to 2020 due to the limited data availability.

### Conventional drought indices

**Figure 5:**
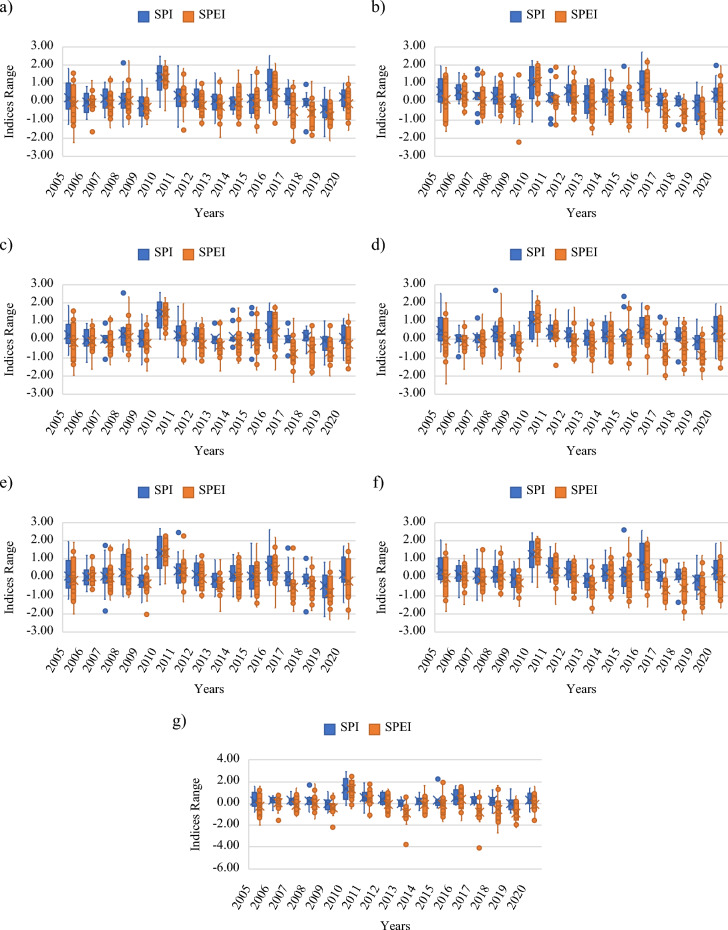
Temporal variations in the SPI and SPEI of the study area between 2005 and 2020: (**a**) Alice Springs Airport, (**b**) Yambah, (**c**) Undoolya, (**d**) Todd River, (**e**) Garden, (**f**) Bond Springs Homestead, and g) Allambi.

As mentioned in the Methodology section, seven meteorological stations from Alice Springs were used to calculate the SPI, SPEI, PDSI, PNI, CZI, MCZI, RAI, RDI, and ZSI. The PET (I3) computation is considered essential for calculating RDI and SPEI. To compute I3 values, precipitation and temperature data (I1 and I2) spanning from 1985 to 2020 were used for the selected meteorological stations. These values of I3, in conjunction with I1 and I2, were then employed to calculate conventional drought indices and to construct the AI models. Fig. 5 illustrates the temporal fluctuations in the SPI and SPEI values for each station across the period from 2005 to 2020. The SPI and SPEI values frequently changed; that is, the drought conditions fluctuated throughout the study period. The correlation coefficient between the two indices was 0.81; this high correlation indicates a strong agreement between SPI and SPEI within their similar index range of -3.0 to 3.0; corroborating findings reported in previous research^37^.

Table 2 lists the minimum and maximum SPI and SPEI outcomes for all stations. The SPI and SPEI classification criteria imply negative and positive values denote dry and wet conditions, respectively. It can be observed that all stations, except the Allambi station, had similar SPI and SPEI values with slight differences. The Allambi station demonstrated the most extreme conditions in September 2010, with a maximum SPI value of 2.91, and minimum and maximum SPEI values of − 4.07 in July 2017 and 2.60 in September 2010, respectively. Moreover, the extreme dryness condition (in terms of the SPI) was found in the Garden station in December 2019 with a value of -2.17. Detailed monthly results of all drought indices can be found in the supplementary files.

**Table 2:** Minimum and maximum SPI and SPEI values for the chosen stations.

Station	SPI		SPEI	
	Minimum	Maximum	Minimum	Maximum
Alice Springs Airport	2.54	− 1.92	2.24	− 2.25
Yambah	2.75	− 1.27	2.41	− 2.22
Undoolya	2.58	− 1.38	2.36	− 2.35
Todd River	2.70	− 1.22	2.52	− 2.46
The Garden	2.69	− 2.17	2.29	− 2.33
Bond Springs Homestead	2.61	− 1.38	2.23	− 2.36
Allambi	2.91	− 0.86	2.60	− 4.07

**Figure 6:**
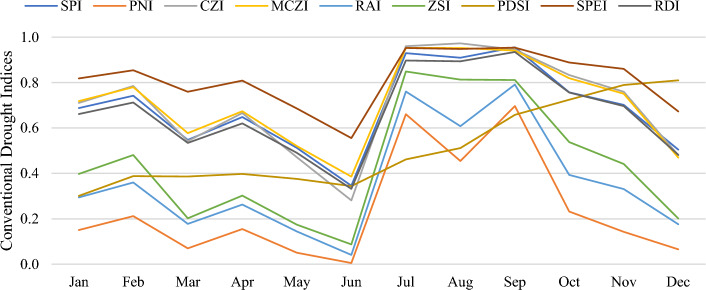
Normalized conventional drought indices of the Garden station for 2010.

The results obtained for the other drought indices can be found in the supplementary files. The indices consistently indicated similar extreme values during drought periods, with particularly notable instances occurring in 2009 and 2019. Figure 6 depicts the fluctuations of the computed normalized conventional drought indices for the Garden station in 2010. Across the various stations addressed in the study, the overall results of all indices showed only slight variations, pointing to uniformity in detecting drought conditions irrespective of the location. The Garden and Allambi stations were identified as the driest and wettest stations, respectively. Moreover, it was found that the RDI had similar behavior to the SPI and SPEI. This is in line with the findings of Tefera et al. (2019), who indicated that these three indices are usually within the same range^21^. On the other hand, the CZI and MCZI demonstrated slight discrepancy as the former used the mean precipitation, whereas the latter considered the median precipitation.

Afterward, to investigate the best-performing conventional drought index, a comparative assessment was applied based on the correlations among the conventional indices and the five selected drought indicators. Table 3 presents the correlations between each conventional drought index and the drought indicators, arranged in a color-coded format that progresses from red to green to indicate increasing values. The Pearson correlation coefficients were calculated across 1344 data points, derived from data spanning 7 stations, 12 months, and 16 years. The RAI displayed the strongest correlations with DI4, DI3, and DI2, with coefficients of 0.718, 0.620, and 0.603, respectively, whereas the PDSI correlated most with DI1, achieving a coefficient of 0.596. In contrast, DI5 had the lowest correlations with all the indices, with PDSI scoring the lowest coefficient of 0.543. Furthermore, the SPEI demonstrated better correlations than the SPI with all indicators, except for DI4. This could be attributed to the incorporation of temperature and PET into the SPEI computation, which was absent in the SPI calculation. This agrees with the outcomes of Vicente-Serrano et al. (2012), who argued that the SPEI has an improved potential to identify drought impacts^38^. These findings agree with those of the studies in the literature that used similar assessment approaches^39,40^. Overall, it can be concluded that the RAI was the most correlated conventional drought index with the drought indicators; hence, it was the most suitable conventional drought index for the study area.

**Table 3:**
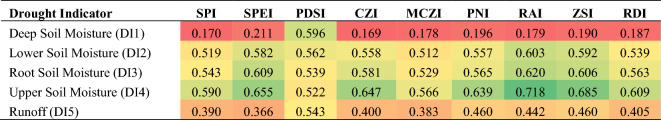
Pearson correlation coefficients between the drought indices and indicators.

### Soft computing models

Six different AI models, as defined in the Methodology section, were utilized in this study to assess and evaluate the drought in Alice Springs using RapidMiner software. The discussion of the AI-based drought indices begins with a description of the output that was used with the meteorological inputs for model training. Next, the developed AI models are tested and correlated with the conventional drought indices and indicators.

#### Model outputs

The supervised AI techniques used in this study required a set of inputs and outputs for their training processes; the inputs constituted three parameters (rainfall, maximum/mean temperature, and PET), whereas the output was taken as the average of the normalized best-performing conventional indices. Each conventional drought index has different ranges and classifications. Therefore, prior to taking the average of the best-performing conventional indices, the output drought levels had to be normalized. This normalization step made all outputs consistent within the range of 0 to 1. The three best conventional indices that had the highest correlations with the drought indicators were the RAI, ZSI, and SPEI, with coefficients of 0.718, 0.685, and 0.655, respectively. The normalized outputs were averaged over the three best-performing indices and used to train the AI models. It was hypothesized that an AI model trained on such a hybrid dataset could potentially outperform all the conventional indices used in its development.

#### Artificial intelligence-based drought assessment

Six AI-based drought indices were developed using DT, GLM, SVM, ANN, DL, and RF models. To assess the performance of the examined machine learning algorithms, two statistical parameters were calculated, the RMSE and R^2^, as listed in Table 4. The SVM outperformed the other models, with RMSE and R^2^ values of 0.031 and 0.951, respectively. This could be attributed to its ability to recognize and incorporate support vectors while training and prevent non-support vectors from impacting the model. This led the model to accommodate noisy conditions^41^. Moreover, another key feature of the SVM is that its input vectors are quite flexible, facilitating the integration of other external parameters, including temperature, rainfall, and wind speed, into the model^42^. Furthermore, the RF and DL models yielded high performance with RMSEs of 0.034 and 0.036, respectively. This was due to their developed ability to capture non-linear relationships between variables. On the other hand, the GLM achieved the lowest accuracy, with RMSE and R^2^ values of 0.054 and 0.856, respectively.

**Table 4:** Comparative assessment of different machine learning techniques.

Evaluation criteria	Decision tree	Generalized linear model	Support vector machine	Artificial neural network	Deep learning	Random forest
R^2^	0.940	0.856	0.951	0.935	0.937	0.945
RMSE	0.036	0.054	0.031	0.044	0.036	0.034

Table 5 showcases the Pearson correlation coefficients between each conventional drought index and the outcomes of the developed AI-based indices. The table is visually arranged with a color-coded scheme that represents the correlation values in ascending order, transitioning from red for lower values to green for higher values, indicating the strength of the relationships. The DT-based index achieved the highest correlation, scoring 0.972 with RAI, whereas the lowest correlation was found to be 0.571 between GLM and PDSI. These findings indicate that the examined AI-based indices were capable of adequately predicting drought levels, particularly in the study area, at a monthly timescale.

**Table 5:**
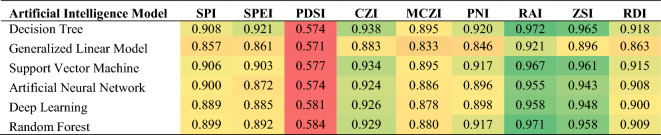
Correlations between the artificial intelligence models and the conventional drought indices.

**Figure 7:**
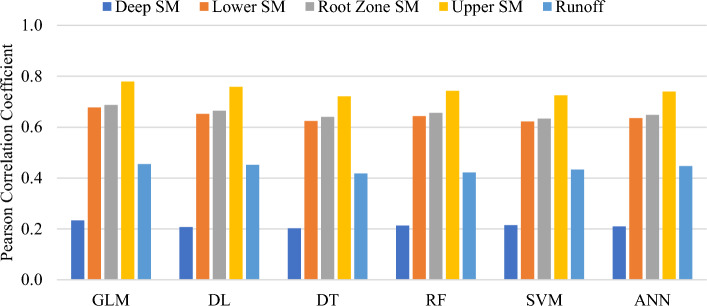
Correlations of the drought indicators with several machine learning models: a decision tree (DT), a generalized linear model (GLM), a support vector machine (SVM), an artificial neural network (ANN), a deep learning (DL) model, and a random forest (RF).

The AI models' performance was validated through the calculation of their Pearson correlation coefficients with various drought indicators. Figure 7 depicts the correlation between the six AI-based indices and the five indicators. The table revealed that all indices exhibited similar behavior in relation to the drought indicators. Overall, the highest correlation was between DI4 and the AI-based indices, followed by DI3; whereas DI1 and DI5 demonstrated the lowest correlations. Similarly, Tian et al.^43^ proposed a hybrid drought index that demonstrated a high correlation with soil moisture at a 5-cm depth. The highest correlation recorded was 0.779 between the GLM index and DI4, while the DT index and DI1 had the lowest correlation at 0.203.

As explained in the introduction, only a few studies studied multiple artificial techniques for drought assessment. For instance, Mokhtarzad et al.^44^ described that an SVM model provides more accurate forecasting values than ANN and ANFIS, with a correlation coefficient of 0.9974. Moreover, using the SPI as an output for developing an AI drought model is a deficiency, as it will not produce a better prediction than the SPI. However, in the current study, different numbers of inputs were tested, and the outputs of the AI models were tested as the average normalized values of the top-performing conventional indices. Additionally, multiple AI models were established, and their validity was ascertained based on their correlation with various drought indicators. In another study, Khan et al. (2020) showed that the SVM demonstrated the highest correlation with a value of 0.94, followed by the ANN (0.87) and KNN (0.85)^24^. Overall, models based on SVM were more adept at capturing the temporal and spatial characteristics of drought compared to those based on ANN and KNN. The KNN model, being applied to drought modeling for the first time, did not perform as well as SVM and ANN models, potentially due to SVM's superior generalization capabilities. In this study, the correlations between the developed models and drought indicators were deduced, not only the drought indices, showing that the GLM was highly correlated with DI4 with a value of 0.779. Also, more soft computing techniques than any other study in the literature were studied in this study.

### Remarks, limitations, and future prospects

**Figure 8:**
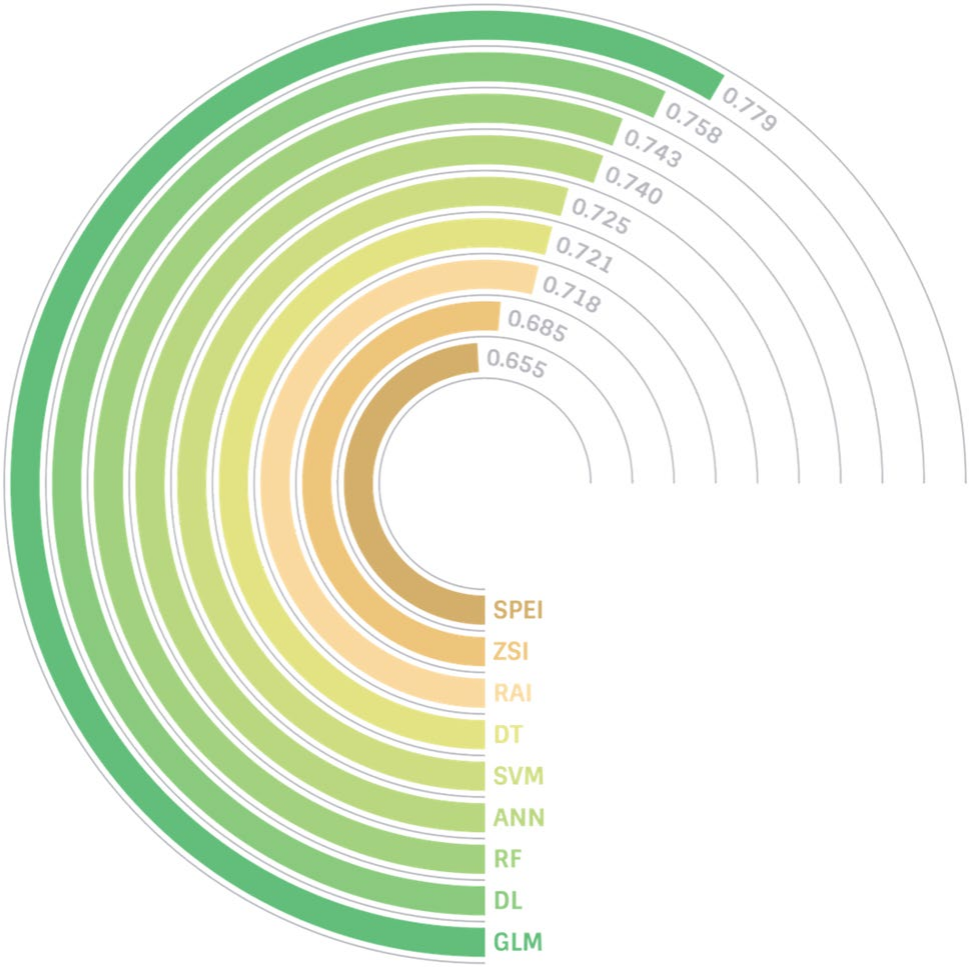
Combined results of the correlations with the upper soil moisture for all examined drought indices.

The present study aimed to develop a novel meteorological drought index by investigating multiple conventional and soft computing algorithms. To summarize the most significant findings, Fig. 8 compares the correlation results with DI4 for the top three performed conventional drought indices and AI-based indices. DI4 was selected since it was the highest correlated index with most drought indices, as concluded throughout the discussion. Overall, all the developed AI-based indices clearly outperformed the conventional indices in terms of drought forecasting, with the GLM-based index achieving the best performance. Significant differences were observed among the correlation results of the selected conventional indices, whereas those of the AI-based model slightly differed.

The obtained results highlighted the robustness of the tested AI models in forecasting and assessing drought. However, the main limitation of this study is that the AI models were trained, in the best-case scenario, to perform as well as the conventional indices; i.e., an AI model could not outperform the conventional index used to train it. To overcome this drawback, this study used an average normalized set of top indices, selected based on their correlations with drought indicators, rather than limiting the methods to drought indices. This is similar to AI, which requires massive training data for forecasting unfamiliar meteorological conditions. In future work, the scope of this study could be extended by applying the developed AI-based indices to other regions throughout the world in which data are available under various characteristics and different features. With the application of the newly developed indices, projections for the near and far future—considering climate change patterns—can be established for drought assessment and forecasting. Furthermore, a more comprehensive range of soft computing models can be investigated.

## Conclusion

The present study aimed to develop novel drought indices based on advanced soft computing models. A comparative assessment was carried out between the developed AI-based indices and nine conventional drought indices according to their correlations with multiple drought indicators. Several AI-based models, including DT, GLM, SVM, ANN, DL, and RF, were implemented to assess drought in Alice Springs, Australia. Various climatic data acquired from available rainfall stations between 1985 and 2020 were used to calculate the conventional drought indices at seven meteorological stations. Also, several drought indicators were collected over the period from 2005–2020 and were utilized as the basis for the comparison between the conventional and AI-based drought indices.

All machine learning algorithms achieved high testing accuracies, where SVM had the lowest RMSE of 0.031, followed by RF and DL with RMSEs of 0.034 and 0.036, respectively. The developed AI-based indices were assessed by computing their Pearson correlation coefficients with both the conventional indices and drought indicators. The highest correlation was 0.972 between the DT and RAI, while the lowest correlation was 0.571 between the GLM and PDSI. Furthermore, the GLM achieved the best performance in terms of its correlations with drought indicators, with a coefficient of 0.778 for DI4. Overall, the findings proved that soft computing models could be considered robust approaches for the rapid and accurate modeling of drought. This paper presented novel high-performing drought indices that can provide decision-makers with a reliable tool for drought management and monitoring.

### Supplementary Information

Supplementary Information 1.

Supplementary Information 2.

## Data Availability

The data used in this study are available online from the Australian Bureau of Meteorology, using this link: http://www.bom.gov.au/climate/data/index.shtml?bookmark=136.

## Abbreviations

AI: Artificial intelligence
ANFIS: Adaptive neuro-fuzzy inference system
ANN: Artificial neural network
CANFIS: Co-active neuro-fuzzy inference system
CZI: China-Z Index
DL: Deep learning
DT: Decision trees
GHG: Greenhouse gases
GLM: Generalized linear model
IDW: Inverse distance weighting
KNN: K-Nearest neighbour
MCZI: Modified China-Z Index
MLP: Multilayer perceptron
PDSI: Palmer Drought Severity Index
PET: Potential evapotranspiration
PNI: Percent of Normal Index
R^2^: Coefficient of determination
RAI: Rainfall Anomaly Index
RDI: Reconnaissance Drought Index
RF: Random forest
RMSE: Root mean square error
scPDSI: Self-calibrating PDSI
SPEI: Standardized Precipitation Evapotranspiration Index
SPI: Standardized Precipitation Index
SVM: Support vector machine
ZSI: Z-Score Index
