# Supplementary material for: Drought prediction using artificial intelligence models based on climate data and soil moisture
Source: Sci Rep. 2024 Aug 24;14:19700. doi: 10.1038/s41598-024-70406-6 (PMC11344829; doi:10.1038/s41598-024-70406-6)
Supplement: Supplementary file 1 — Supplementary Information 1. [file 41598_2024_70406_MOESM1_ESM.docx]

**Supplementary Files**

# Conventional Drought Indices

Multiple conventional drought indices, particularly standardized precipitation index (SPI), standardized precipitation index (SPEI), Palmer drought severity index (PDSI), percent of normal index (PNI), China-Z index (CZI), modified China-Z index (MCZI), rainfall anomaly index (RAI), Z score index (ZSI), and reconnaissance drought index (RDI), were used in this study.

## Standardized Precipitation Index

SPI was designed by members of Colorado State University in 1993 as a simple, practical method for monitoring drought [1]. The index is widely used in several drought-related applications around the world due to its flexibility and dependence only on rainfall [2]. On the other hand, the accuracy of measurements, the number of gauges, and the length of the record are major factors that could negatively affect the quality of precipitation data. Another disadvantage is using only precipitation records in index calculations, excluding climate variables, such as air temperature and humidity [3].

## Standardized Precipitation Evapotranspiration Index

The SPEI was developed by Vicente-Serrano from the Pyrenean Institute of Ecology in Zaragoza, Spain. Drought-related conditions associated with various drought impacts can be identified, monitored, and analyzed using this method. SPEI uses SPI as its basis but involves a temperature component, which enables it to measure drought severity according to intensity and duration, identify the onset and termination of the drought period, and consider the effect of temperature on the drought development [4]. Regardless of the common use of the SPI index because of its ease, precipitation is the only input required for its drought forecasting model; other atmospheric conditions must be considered as they impact frequency and magnitude (e.g., precipitation, temperature, and evapotranspiration). The SPEI was developed to ensure that these atmospheric elements were included in drought forecasts, particularly potential evapotranspiration. The SPEI is derived from precipitation and potential evapotranspiration (PET) data. The PET was calculated as follows:

|  | $PET = {16K(\frac{10T}{I})}^{m}$ | (1) |
| --- | --- | --- |

where *T* is the average monthly temperature (°C), *m* is the equivalent to 6.75 x 10^-5^, *I* is the heat index that is calculated as the sum of 12-monthly index values *i*, and *i* is derived from monthly average temperature as shown below:

|  | $i = {16K(\frac{T}{5})}^{1.514}$ | (2) |
| --- | --- | --- |

Moreover, K is the correction coefficient which is derived from the latitude:

|  | $K = (\frac{N}{12}) (\frac{NDM}{30})$ | (3) |
| --- | --- | --- |

where *N* is the maximum number of sun hours and *NDM* represents the number of days in a specific month. The N was calculated as follows:

|  | $N = (\frac{24}{\pi}) \omega$_s_ | (4) |
| --- | --- | --- |

where $\omega$*_s_* is the hourly angle of the sun.

|  | $\omega$_s_ = arccos(-tan$\theta$tan$\delta)$ | (5) |
| --- | --- | --- |

where $\theta$ represents the latitude (radians) and $\delta$ is the declination angle of the sun (radians). In a climatic system, SPEI is the balance between seasonal precipitation (P) and temperature (T) in a given month (i), i.e., D_i_ = P_i_ - PET_i_. The D_i_ can be calculated on varying time frames, and the log-logistic method was used as the statistical distribution method for standardizing the variable to model the D_i_ series. The probability density function (*f(x))* of a three-parameter log-logistic distributed variable was expressed as follows:

|  | $f(\chi)=\frac{\beta}{\alpha}({\frac{\chi-\gamma}{\alpha})}^{\beta-1}(1+({{\frac{\chi-\gamma}{\alpha})}^{\beta})}^{-2}$ | (6) |
| --- | --- | --- |

where α represents scale, β signifies the shape parameter, and γ is the origin variable, respectively, where γ < D < ∞. To obtain the $\alpha$, $\beta$ and $\gamma$ parameters, different procedures can be followed, and the probability-weighted moments (PWMs) method was found to be the most robust and direct approach. The equations below show the formulas utilized to calculate the log-logistic-based parameters ($\alpha$, $\beta$ and $\gamma).$

|  | $\beta=\frac{2W_{1}-W_{O}}{6W_{1}-W_{O}-6W_{2}}$ | (7) |
| --- | --- | --- |
|  | $\alpha=\frac{(W_{O}-2W_{1})\beta}{\Gamma(1+\frac{1}{\beta}) \Gamma(1-\frac{1}{\beta})}$ | (8) |
|  | $\gamma=W_{O}- \alpha\Gamma(1+\frac{1}{\beta}).\Gamma(1-\frac{1}{\beta})$ | (9) |

where Γ(β) represents the gamma function of *β* and *W_s_* coefficient of order s was calculated as follows:

|  | $\omega_{s} = \frac{1}{N}\sum_{i=1}^{N} {(1-F_{i})}^{s}D_{i}$ | (10) |
| --- | --- | --- |

where *N* is the number of data points and *F_i_* refers to the frequency estimator based on [5], which was calculated as follows:

|  | $F_{i} = \frac{i - 0.35}{N}$ | (11) |
| --- | --- | --- |

where *i* is the number of observations in ascending order. The probability distribution function of the *D* series based on the log-logistic distribution was calculated according to Equation 12, and SPEI was calculated as the standardized value of F(x) as shown in Equation 13:

|  | $F(\chi) = {(1+{(\frac{\alpha}{\chi-\gamma})}^{\beta})}^{-1}$ | (12) |
| --- | --- | --- |
|  | $\mathrm{SPEI}=\sqrt{-2ln(P)}-\frac{C_{O}+C_{1}W+ C_{2}W^{2}}{1+d_{1}W+ d_{2}W^{2}+ d_{3}W^{3}}$ | (13) |

where *P* is the probability of exceeding a predetermined D value (*P*=1-F(x)) in which *P* is replaced by 1− *P* and the sign of the SPEI is reversed when P > 0.5. Moreover, *C_0_* is the coefficient with a value of 2.515517, C_1_ is 0.802853, C_2_ is 0.010328, d_1_ is 1.432788, d_2_ is 0.189269, and d_3_ is 0.001308.

## Palmer Drought Severity Index

The Palmer Index was developed by Palmer in 1965 as one of the first attempts to detect droughts based on precipitation and temperature data. The PDSI is an index of meteorological drought based on precipitation and temperature data as well as information on the water-holding capacity of the soil. Taking into consideration the received precipitation, moisture content stored in the soil, and potential loss of moisture due to high temperature. Palmer (1965) reported that the monthly index time series ranges from -4 to +4 [6]. A negative PDSI indicates a dry period, and a positive indicates a wet period. PDSI has a 9-month timescale. Therefore, it cannot identify droughts over shorter periods.

## Percent of Normal Index

PNI can be used to assess the severity of drought events. This index was found to depict the severity of meteorological droughts. Thus, drought trend analysis using PNI is concluded to be a useful and practical approach. This index was first as a normal precipitation percentage that is simple and easy to calculate [7]. A variety of time scales, from a single month to several months representing a season or water year, can be used to calculate this index. The input of PNI only includes the precipitation values.

## China-Z Index and Modified China-Z Index

This index was developed by the National Climate Centre of China, China in 1995. Similar to SPI, it uses the monthly precipitation to determine wet and dry periods over multiple timescales. The MCZI was calculated by substituting the median precipitation for the mean precipitation in the same equation [8].

## Rainfall Anomaly Index

There are several factors influencing drought in a region, including vegetation status, surface wetness, and temperature; however, anomalous rainfall also affects the storage of soil moisture. Agricultural regions of subtropical semiarid ecosystems are subjected to droughts caused by it. The RAI indicates deviations from the long-term rainfall average [9]. As RAI is flexible, it can be analyzed on various timescales for various types of droughts affecting agriculture, water resources, and other sectors. This method has the advantage of studying the drought index with only one input. The results of drought indices can be presented for different periods, including monthly, seasonal, and annual.

## Z Score Index

ZSI index is a dimensionless parameter that utilizes the original precipitation data similar to SPI. Neither the type III nor the gamma distribution of the precipitation data are necessary for ZSI [10]. Similar to CZI, ZSI is also capable of accommodating missing data.

## Reconnaissance Drought Index

As a general meteorological indicator, RDI provides information about drought severity and duration and considers the water deficit as a balance between input and output in a water system [11]. The RDI appears to represent the drought conditions more realistically. When the PET is integrated with precipitation, it can effectively be used to measure droughts in areas with different climatic conditions. In contrast to other indices, the RDI offers universal applicability as it is based on precipitation and potential evapotranspiration [12].

## Summary

Table S1 lists the input parameters, along with classifications and ranges for the drought indices.

Table S1: Input parameters and classifications of the conventional drought indices.

| Drought index | SPI | PNI | CZI | MCZI | RAI | ZSI | PDSI | SPEI | RDI |
| --- | --- | --- | --- | --- | --- | --- | --- | --- | --- |
| Reference | [1] | [7] | [8] | [8] | [9] | [10] | [6] | [4] | [13] |
| Input parameters | P | P | P | P | P | P | P, T, AWC, L | P, T, and PET | P, T, and PET |
| Classification & ranges* | Extremely wet (≥ 2) | Wet (≥ 120) | Extremely wet (≥ 2) | Extremely wet (≥ 2) | Extremely wet (≥ 3) | Extremely wet (≥ 2) | Extremely wet (≥ 4) | Extremely wet (≥ 2) | Extremely wet (≥ 2) |
|  | Very wry (1.5-1.99) | Normal (80-120) | Very wet (1.5-1.99) | Very wet (1.5-1.99) | Very wet (2-2.99) | Very wet (1.5-1.99) | Very wet (3-3.99) | Very wet (1.5-1.99) | Very wet (1.5-1.99) |
|  | Moderately wet (1-1.49) | Slightly dry  (70-80) | Moderately wet (1-1.49) | Moderately wet (1-1.49) | Moderately wet (1-1.99) | Moderately wet (1-1.49) | Moderately wet (2-2.99) | Moderately wet (1-1.49) | Moderately wet (1-1.49) |
|  | Normal (0.99-0) | Moderately dry (55-70) | Normal (0.99-0) | Normal (0.99-0) | Slightly wet (0.5-0.99) | Normal (0.99-0) | Slightly wet (1-1.99) | Normal (0.99-0) | Normal (0.99-0) |
|  | Near normal (0- -0.99) | Severely dry (40-55) | Near normal (0- -0.99) | Near normal (0- -0.99) | Near normal (-0.49-0.49) | Near normal (0- -0.99) | Incipient wet spell (0.5-0.99) | Near normal (0- -0.99) | Near normal (0- -0.99) |
|  | Moderately dry (-1- -1.49) | Very severe dry (≤ 40) | Moderately dry  (-1- -1.49) | Moderately dry (-1- -1.49) | Slightly dry  (-0.99- -0.5) | Moderately dry (-1- -1.49) | Near normal  (-0.49-0.49) | Moderately dry (-1- -1.49) | Moderately dry (-1- -1.49) |
|  | Severely dry (-1.5- -1.99) |  | Severely dry (-1.5- -1.99) | Severely dry (-1.5- -1.99) | Moderately dry  (-1.99- -1) | Severely dry (-1.5- -1.99) | Incipient dry spell (-0.0- -0.99) | Severely dry (-1.5- -1.99) | Severely dry (-1.5- -1.99) |
|  | Extremely dry (≤ -2) |  | Extremely dry (≤ -2) | Extremely dry (≤ -2) | Very dry  (-2.99- -2) | Extremely dry (≤ -2) | Mild dry  (-1- -1.99) | Extremely dry (≤ -2) | Extremely dry (≤ -2) |
|  |  |  |  |  | Extremely dry (≤ -3) |  | Moderately dry (-2- -2.99) |  |  |
|  |  |  |  |  |  |  | Severely dry  (-3- -3.99) |  |  |
|  |  |  |  |  |  |  | Extremely dry (≤ -4) |  |  |

# Conventional Drought Indices Results

Figure S1-8 show the temporal variations of the conventional drought indices between 2005 and 2020 in the study area.


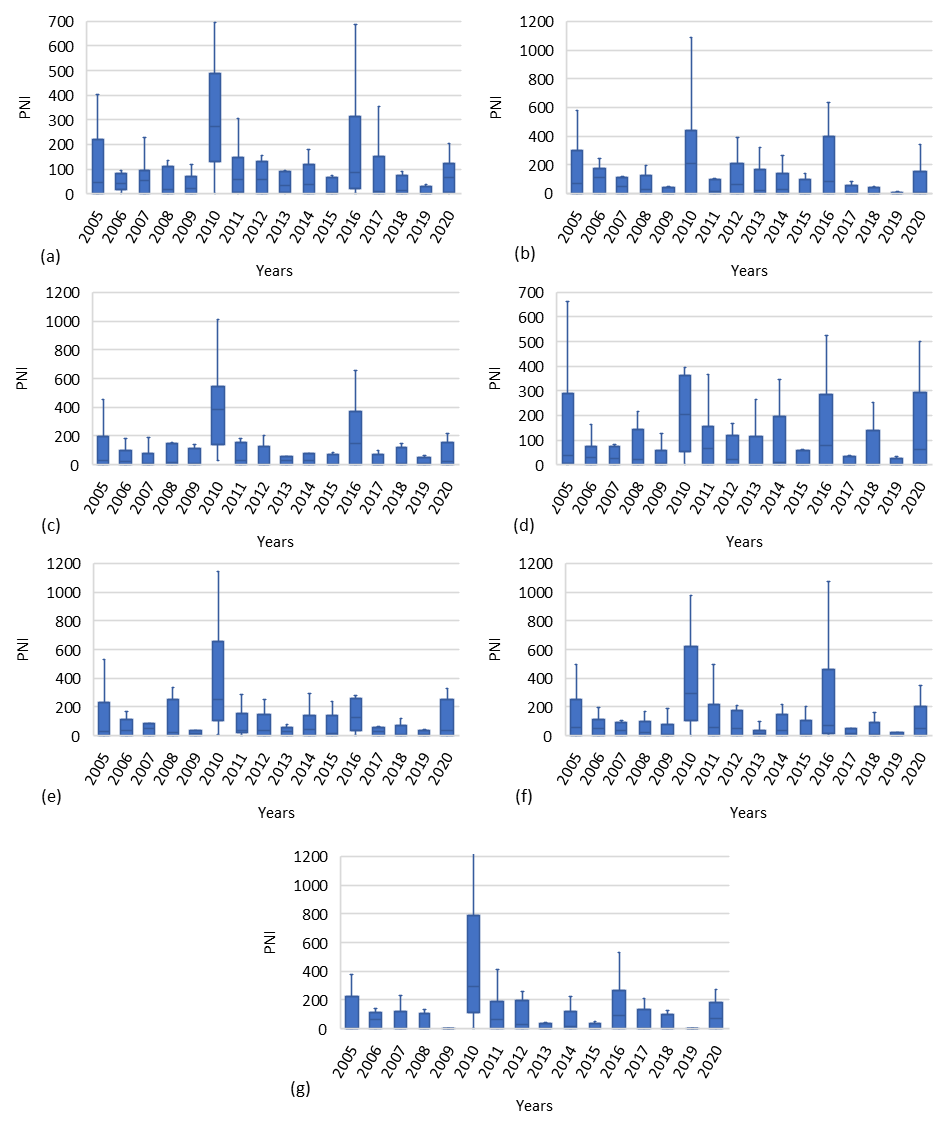


Figure S1: Temporal variations of Percent Normal Index (PNI) between 2005 and 2020 in the study area: a) Alice Springs Airport, b) Yambah, c) Undoolya, d) Todd River, e) The Garden, f) Bond Springs Homestead, and g) Allambi.


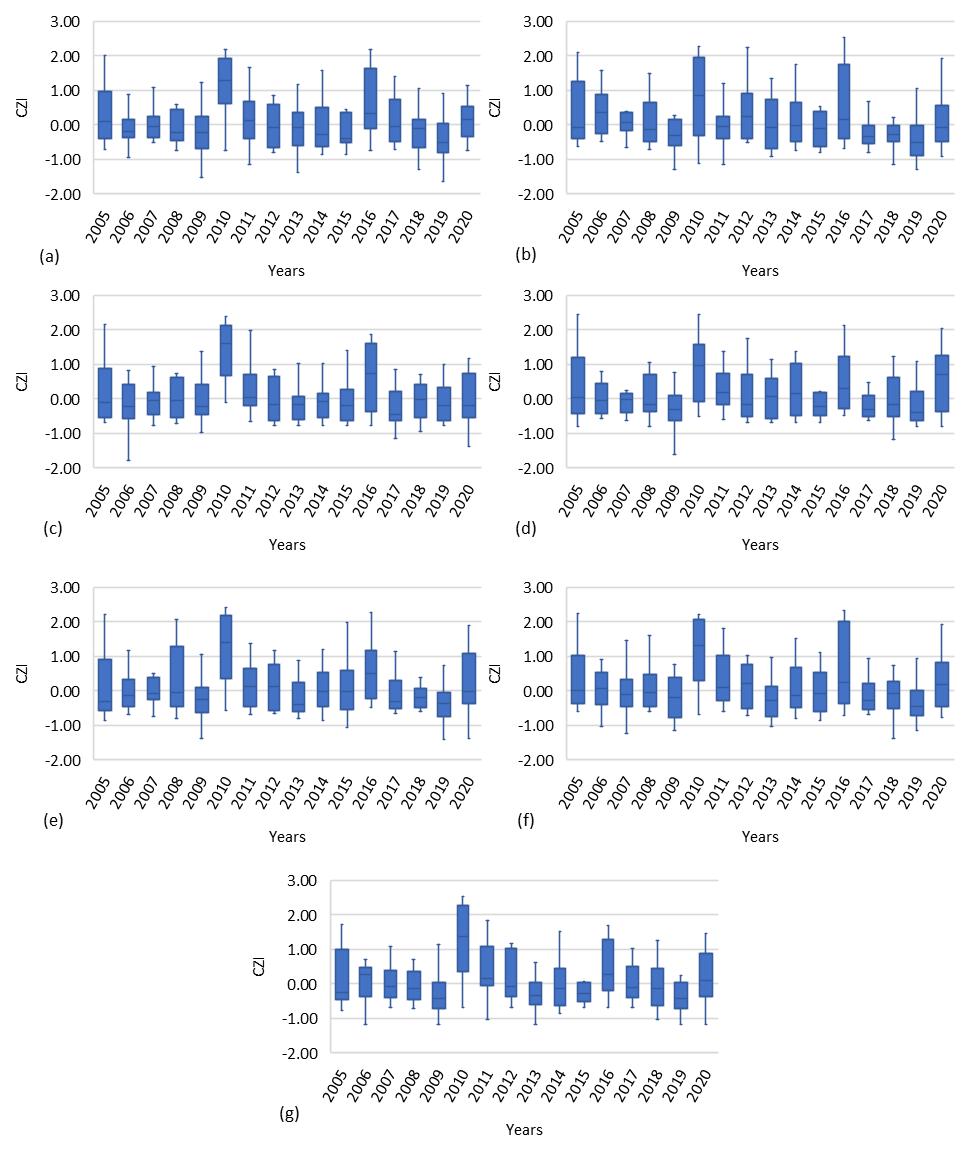


Figure S2: Temporal variations of China Z-Index (CZI) between 2005 and 2020 in the study area: a) Alice Springs Airport, b) Yambah, c) Undoolya, d) Todd River, e) The Garden, f) Bond Springs Homestead, and g) Allambi.


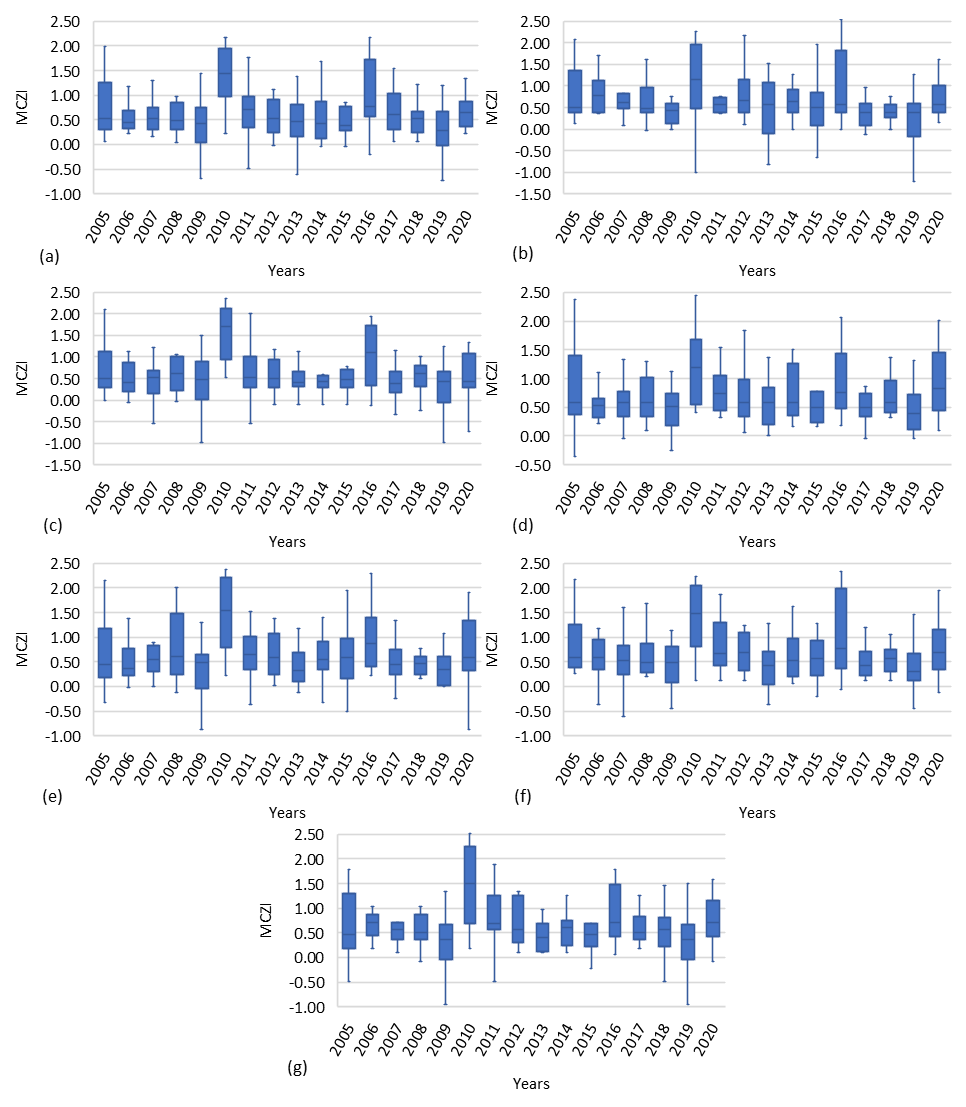


Figure S3: Temporal variations of Modified China Z-Index (MCZI) between 2005 and 2020 in the study area: a) Alice Springs Airport, b) Yambah, c) Undoolya, d) Todd River, e) The Garden, f) Bond Springs Homestead, and g) Allambi.


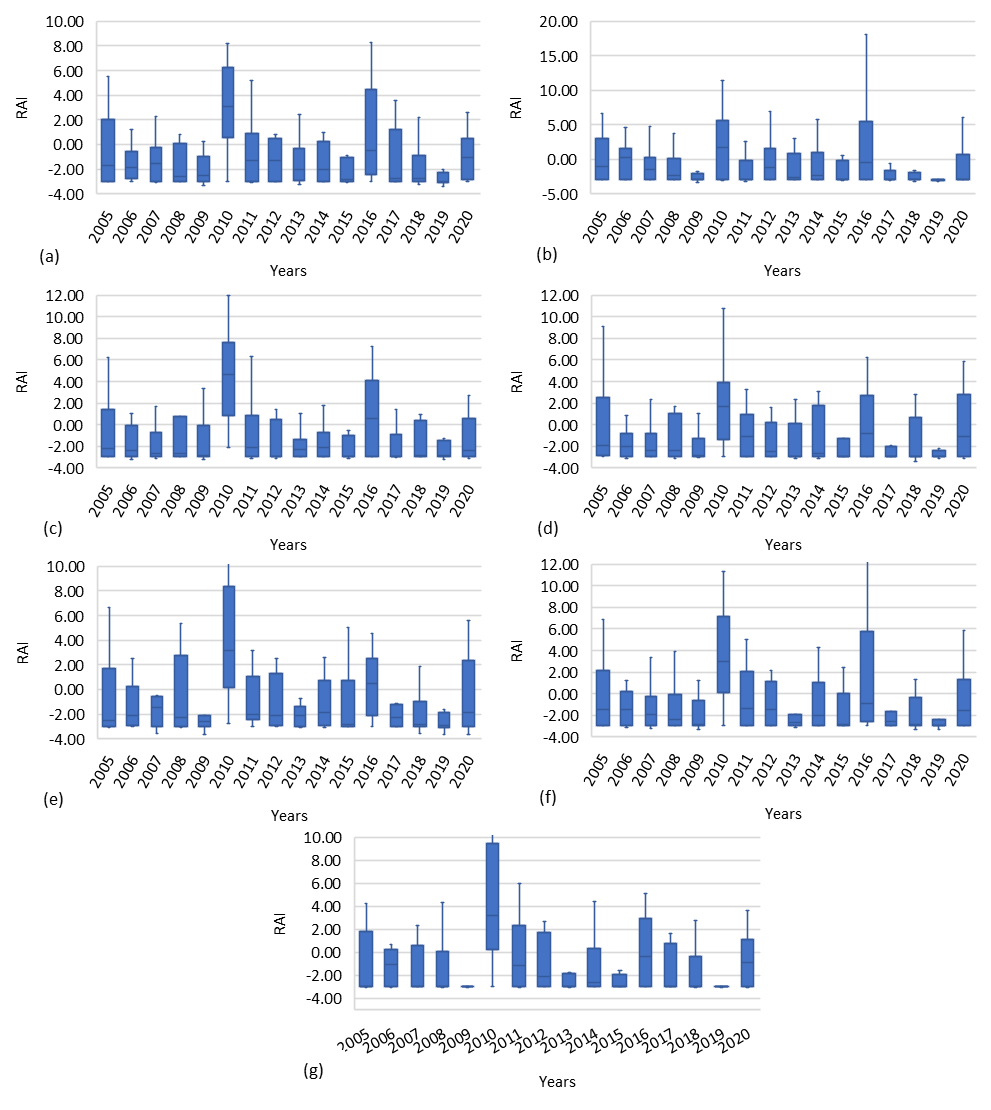


Figure S4: Temporal variations of Rainfall Anomaly Index (RAI) between 2005 and 2020 in the study area: a) Alice Springs Airport, b) Yambah, c) Undoolya, d) Todd River, e) The Garden, f) Bond Springs Homestead, and g) Allambi.


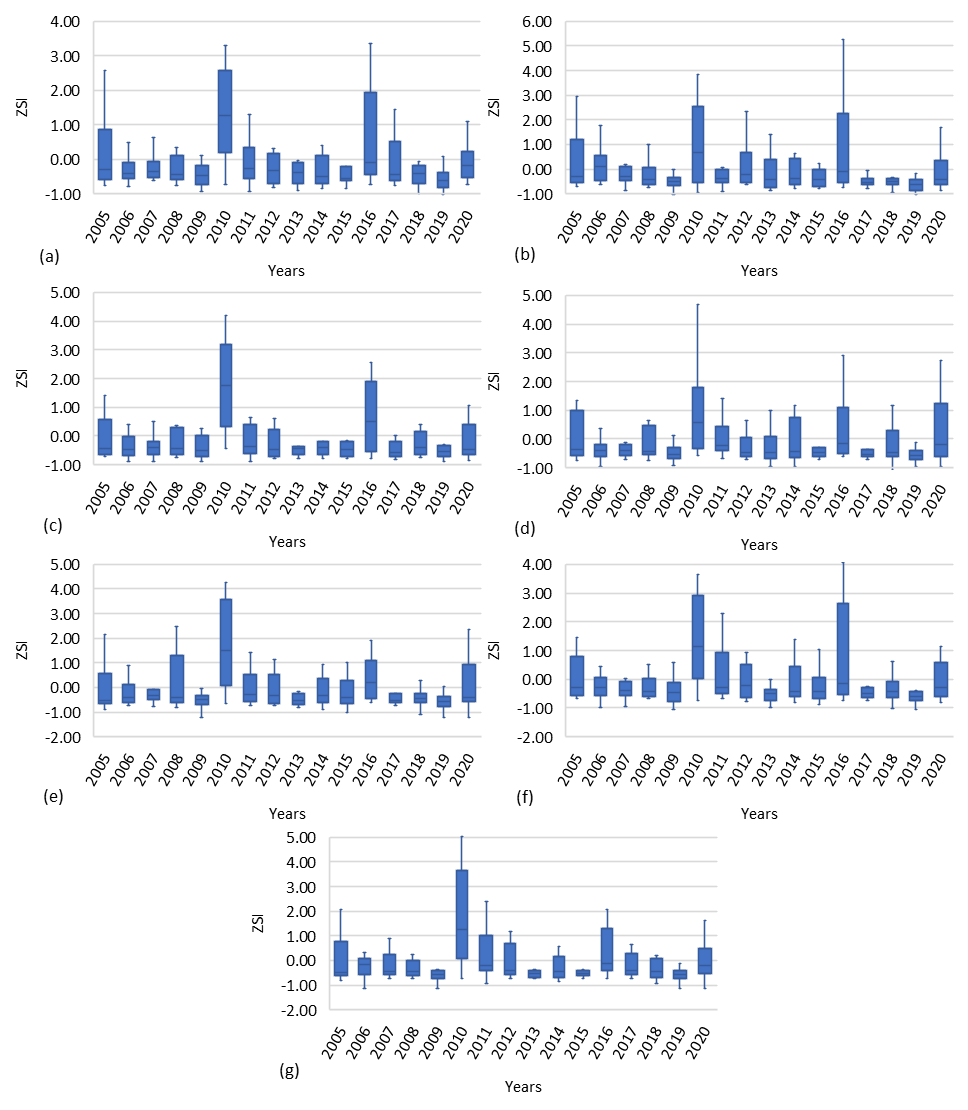


Figure S5: Temporal variations of the Z-Score Index (ZSI) between 2005 and 2020 in the study area: a) Alice Springs Airport, b) Yambah, c) Undoolya, d) Todd River, e) The Garden, f) Bond Springs Homestead, and g) Allambi.


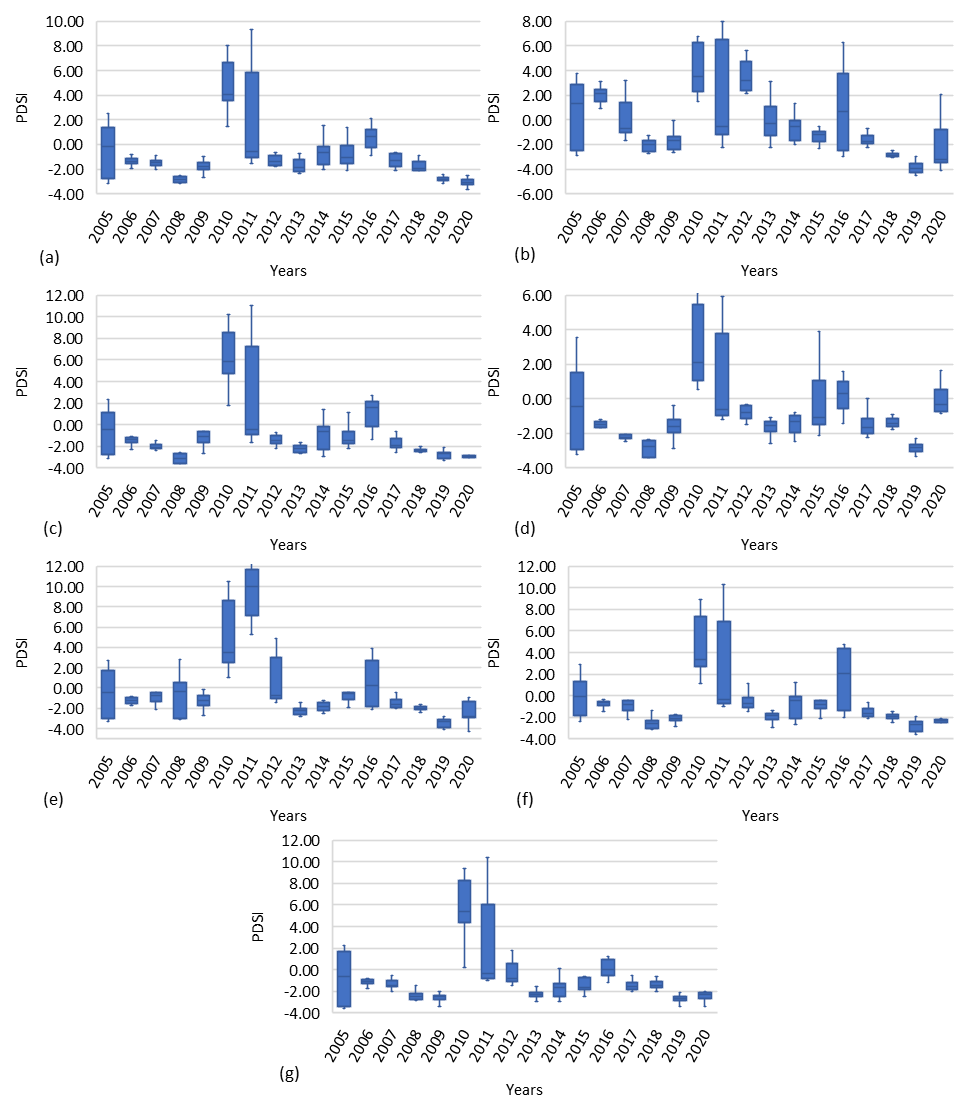


Figure S6: Temporal variations of Palmar Drought Severity Index (PDSI) between 2005 and 2020 in the study area: a) Alice Springs Airport, b) Yambah, c) Undoolya, d) Todd River, e) The Garden, f) Bond Springs Homestead, and g) Allambi.


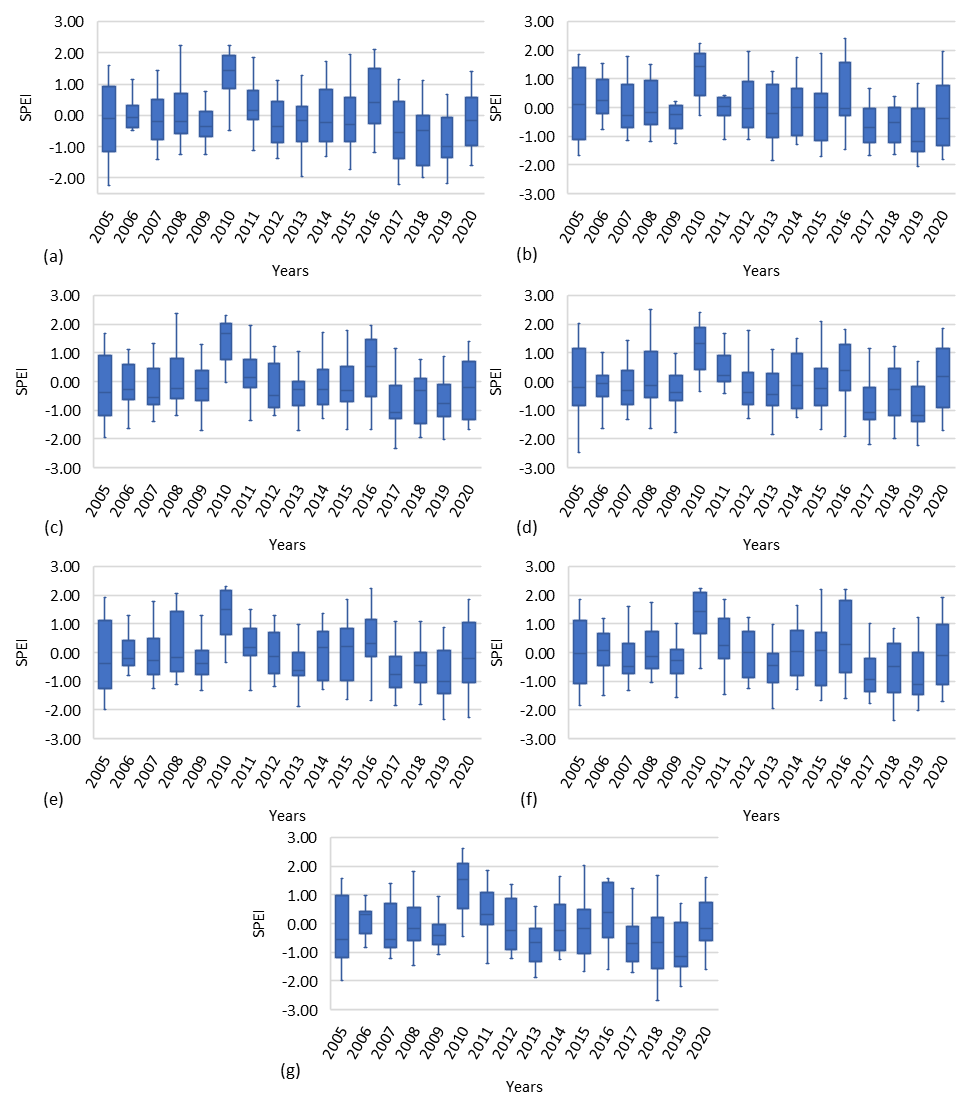


Figure S7: Temporal variations of Standardized Precipitation Evapotranspiration Drought Index (SPEI) between 2005 and 2020 in the study area: a) Alice Springs Airport, b) Yambah, c) Undoolya, d) Todd River, e) The Garden, f) Bond Springs Homestead, and g) Allambi.


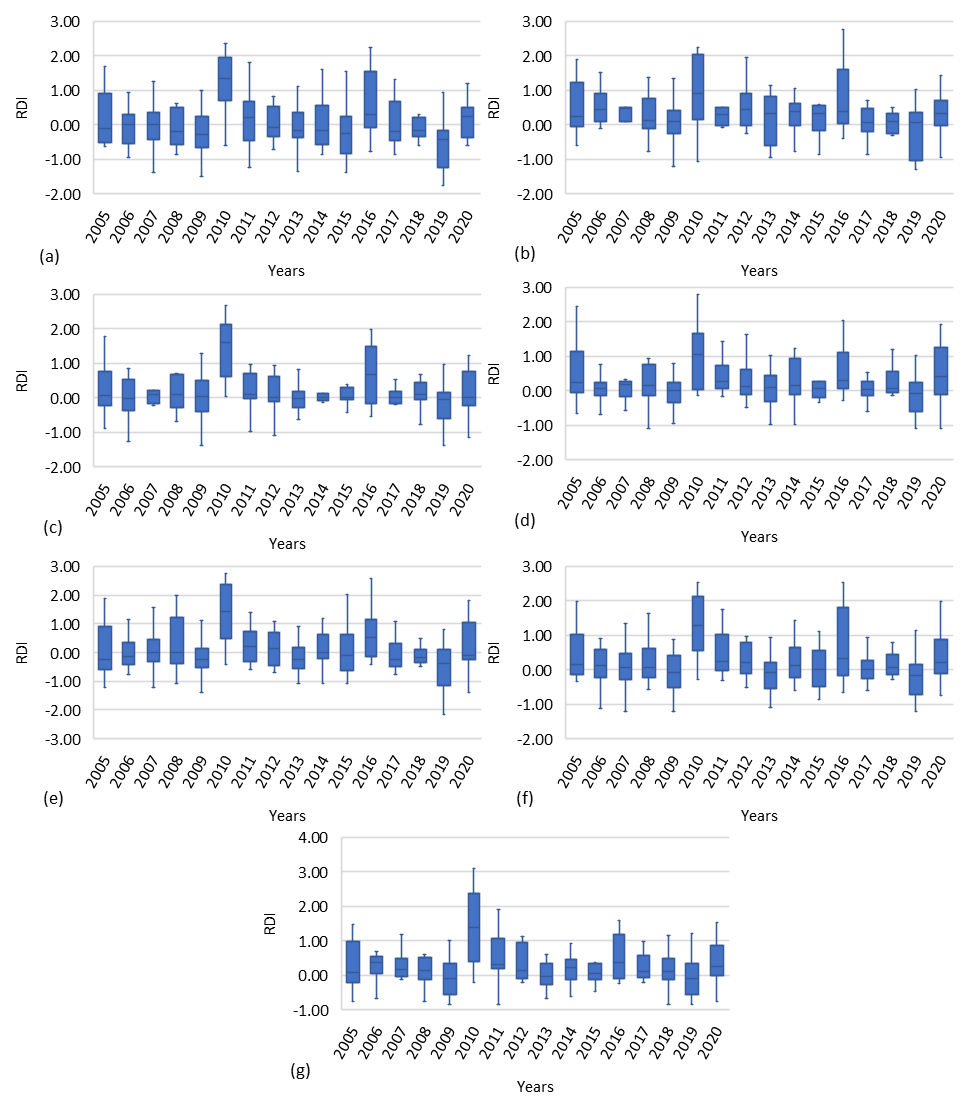


Figure S8: Temporal variations of Reconnaissance Drought Index (RDI) between 2005 and 2020 in the study area: a) Alice Springs Airport, b) Yambah, c) Undoolya, d) Todd River, e) The Garden, f) Bond Springs Homestead, and g) Allambi.

# References

[1] N. J. D. and J. K. Thomas B. McKee, “THE RELATIONSHIP OF DROUGHT FREQUENCY AND DURATION TO TIME SCALES,” *Eighth Conference on Applied Climatology*, no. Jan, p. 6, 1993, doi: 10.1002/jso.23002.

[2] D. Tigkas, “Drought Characterisation and Monitoring in Regions of Greece,” *European Water*, vol. 23/24, no. June, pp. 29–39, 2008.

[3] A. Zargar, R. Sadiq, B. Naser, and F. I. Khan, “A review of drought indices,” *Environmental Reviews*, vol. 19, no. 1, pp. 333–349, 2011, doi: 10.1139/a11-013.

[4] S. M. Vicente-Serrano, S. Beguería, and J. I. López-Moreno, “A multiscalar drought index sensitive to global warming: The standardized precipitation evapotranspiration index,” *J Clim*, vol. 23, no. 7, pp. 1696–1718, 2010, doi: 10.1175/2009JCLI2909.1.

[5] J. R. M. Hosking and J. R. Wallis, “Paleoflood Hydrology and Flood Frequency Analysis,” *Water Resour Res*, vol. 22, no. 4, pp. 543–550, 1986, doi: 10.1029/WR022i004p00543.

[6] W. C. Palmer, “Meteorological Drought,” *U.S. Weather Bureau*. p. 58, 1965. [Online]. Available: https://www.ncdc.noaa.gov/temp-and-precip/drought/docs/palmer.pdf

[7] G. Willeke, J. R. M. Hosking, J. R. Wallis, and N. B. Guttman, “National drought atlas developed,” *Institute for water resources report*, vol. 94, 1994.

[8] M. G. Kendall and A. Stuart, “The Advanced Theory of Statistics,” *Charles Griffin & Company: London, High Wycombe*, vol. 2, 1977.

[9] M. P. Van Rooy, “A rainfall anomaly index independent of time and space,” *Notos*, vol. 14, pp. 43–48, 1965.

[10] M. Triola, “Elementary Statistics,” *Addison-Wesley: Reading*, pp. 691–693, 1995.

[11] M. A. A. Zarch, H. Malekinezhad, M. H. Mobin, M. T. Dastorani, and M. R. Kousari, “Drought Monitoring by Reconnaissance Drought Index (RDI) in Iran,” *Water Resources Management*, vol. 25, no. 13, pp. 3485–3504, 2011, doi: 10.1007/s11269-011-9867-1.

[12] T. Thomas, R. K. Jaiswal, R. V. Galkate, and T. R. Nayak, “Reconnaissance Drought Index Based Evaluation of Meteorological Drought Characteristics in Bundelkhand,” *Procedia Technology*, vol. 24, pp. 23–30, 2016, doi: 10.1016/j.protcy.2016.05.005.

[13] G. Tsakiris and H. Vangelis, “Establishing a Drought Index Incorporating Evapotraspiration,” *European Water*, vol. 9, no. 10, pp. 3–11, 2005.
